# Intraocular infiltration of Philadelphia chromosome-positive acute lymphoblastic leukemia diagnosed by polymerase chain reaction from the aqueous humor: A case report

**DOI:** 10.1097/MD.0000000000018872

**Published:** 2020-01-24

**Authors:** Miki Hiraoka, Hiroshi Ohguro, Hiroshi Ikeda, Daisuke Furuya, Satoshi Takahashi

**Affiliations:** a Department of Ophthalmology; b Department of Oncology and Hematology; c Department of Clinical Engineering, Faculty of Health Sciences, Hokkaido University of Science; d Department of Infection Control and Laboratory Medicine, Sapporo Medical University School of Medicine, Sapporo, Hokkaido, Japan.

**Keywords:** acute lymphoblastic leukemia, hypopyon, Philadelphia chromosome, polymerase chain reaction, Wilms tumor gene

## Abstract

**Rationale:**

Intraocular manifestation of hematopoietic tumors is rare and often difficult to distinguish from inflammation. We report a patient with acute lymphoblastic leukemia (ALL) who developed intraocular infiltration during the remission period.

**Patient concerns:**

A 40-year-old man presented with hypopyon in his right eye. Three months later, extensive subretinal infiltration and the elevation of intraocular pressure were observed. Fourteen months prior to this, he had been diagnosed with Philadelphia chromosome-positive ALL, and had received chemotherapy and bone marrow transplantation that resulted in complete remission.

**Diagnosis:**

The breakpoint cluster region-Ableson (BCR/ABL) chimera was detected by polymerase chain reaction (PCR) analysis in the patient's aqueous humor. Additionally, a high expression of WT1 (Wilms tumor gene) mRNA in the aqueous humor was discovered. A bone marrow examination yielded a high expression of BCR/ABL fusion gene, and it was determined the patient had experienced a relapse of ALL.

**Interventions:**

The dasatinib was administered orally to the patient.

**Outcomes:**

The intraocular infiltration disappeared, and intraocular pressure was normalized.

**Lessons:**

Intraocular infiltration in leukemia patients may be an indication of relapse regardless of systemic conditions. Analyzing mRNA expression of BCR/ABL and WT1 of ocular fluid in patients with hypopyon is beneficial in diagnosing topical relapses in leukemia.

## 1 Introduction

Hypopyon is the accumulation of infiltrating cells in the anterior chamber, and is a sign of uveitis. The 2 major causes of uveitis are autoimmune disease and infection, in both of which inflammatory cells are infiltrated in intraocular fluid. Occasionally, hypopyon is observed in malignant diseases such as hematologic malignant disease and retinoblastoma. The infiltrated cells in such instances contain malignant cells. Distinguishing whether the signs of hypopyon indicate inflammation or malignancy can be difficult.^[1]^ Ocular disorder caused by malignant disease is therefore referred to as a masquerade syndrome that mimics inflammation.^[1]^ The uveitis masquerade syndrome is commonly associated with hematologic malignancies. The major causes of uveitis masquerade syndrome are lymphoma and leukemia.^[1,2]^ The ocular infiltration of malignancy is mainly diagnosed by cytologic examination followed by immunophenotyping using intraocular fluid such as the vitreous body or aqueous humor. Recently, it has been determined that the Wilms tumor gene (WT1) mRNA expression in blood and bone marrow is a prognostic marker in leukemia.^[3]^ However, the WT1 mRNA of the aqueous humor has never been examined.

In this report, we describe a case presenting with unilateral leukemic intraocular infiltration as an initial sign of acute lymphoblastic leukemia (ALL) relapse. The chimeric breakpoint cluster region-Ableson (BCR/ABL) gene in the aqueous humor was detected by polymerase chain reaction (PCR) analysis. Moreover, the aqueous humor showed a high expression of WT1 mRNA. The present findings demonstrate that reveals the chimeric BCR/ABL gene by PCR and detects WT1 mRNA in the aqueous humor.

## 2 Case presentation

The medical records of a patient with intraocular infiltration with acute lymphoblastic leukemia were retrospectively reviewed. The present study protocol was approved by the Ethics Committee of the Sapporo Medical University Hospital and conducted in accordance with the Declaration of Helsinki. After a full explanation of the purpose and protocol for this study, patient has provided informed consent for publication of the case.

### 2.1 Case

Our patient was a 40-year-old man who had developed systemic pain. He had initially visited another hospital 14 months earlier and had been diagnosed with Philadelphia chromosome-positive acute lymphoblastic leukemia (Ph+ALL). At that time, he underwent chemotherapy and bone marrow transplantation and achieved complete remission. However, a month after termination of the chemotherapy, he developed blurred vision in his right eye.

**Figure 1:**
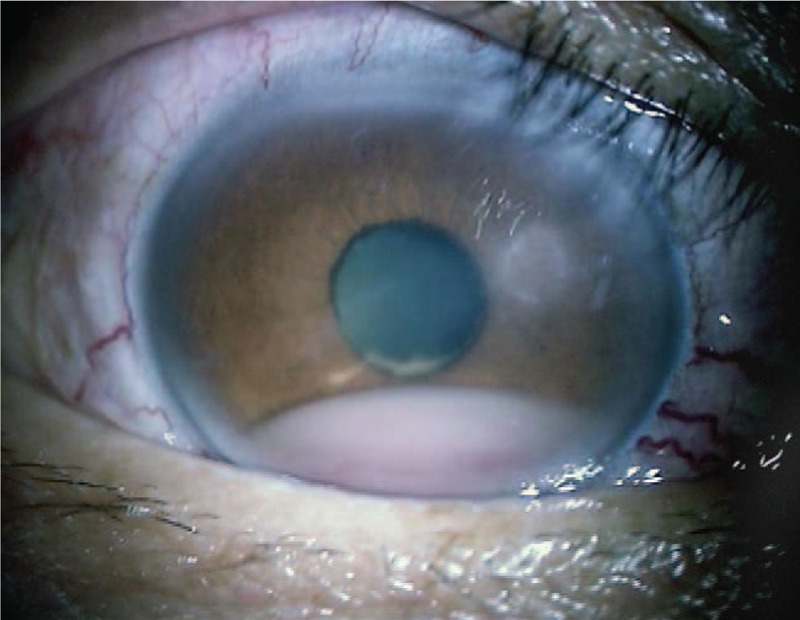
The slit-lamp photographs showed blood-streak hypopyon in the right eye.

**Figure 2:**
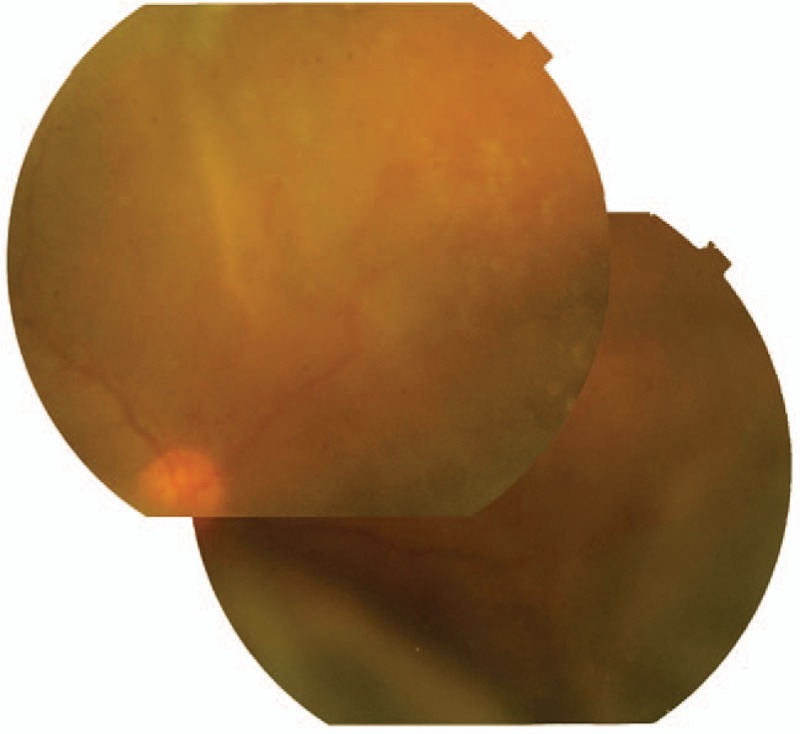
The fundus photographs showed extensive peripheral retinal detachment.

**Figure 3:**
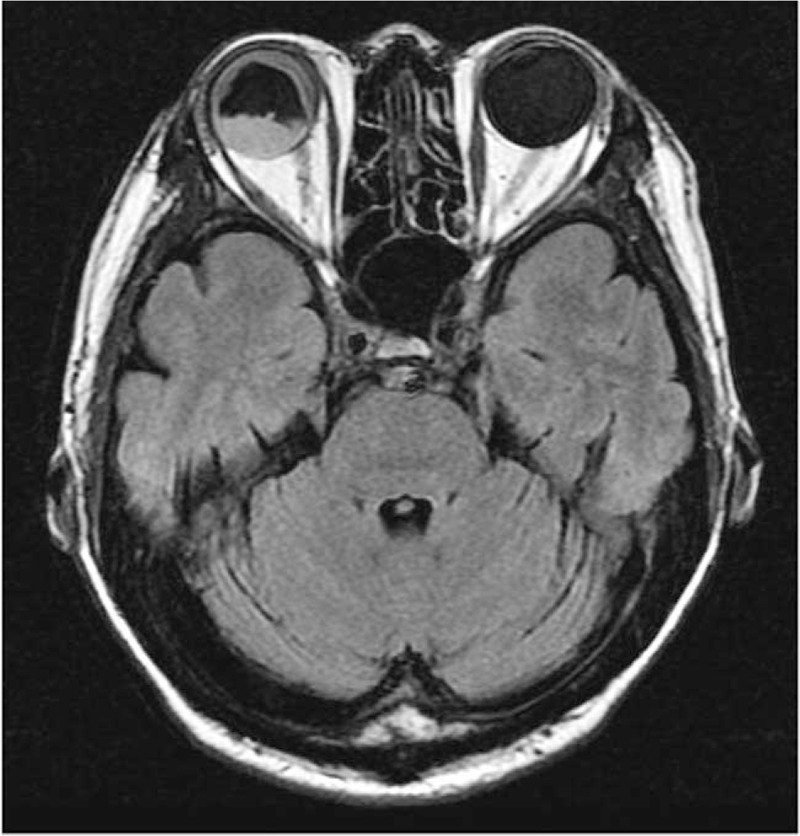
The brain magnetic resonance imaging (MRI) showed a massive high signal lesion in the right eye.

The initial ophthalmic examination disclosed a best-corrected visual acuity (BCVA) of 8/200 in the right eye and 20/12.5 in the left eye. The intraocular pressure (IOP) was 45 mmHg in the right eye and 14 mmHg in the left. A slit-lamp examination demonstrated conjunctival injection, corneal oedema, and hypopyon in the right eye. An ophthalmological examination of his left eye was unremarkable. Results of the laboratory investigations of serum including antinuclear antibodies, anti-double stranded DNA antibodies, rheumatoid factors, and antineutrophil cytoplasmic antibodies were unremarkable. Peripheral blood cell counts were normal. Topical betamethasone and anti-glaucoma medication were applied resulting in normalized IOP to 10 mmHg and improved BCVA to 20/12.5 in his right eye a week later. There was no abnormality in his right eye fundus. Three months following the initial presentation, his right eye developed severe vision loss and ocular pain. Upon examination, the eye showed conjunctival injection, corneal edema, blood-streaked hypopyon, a shallow chamber, and extensive retinal detachment (Figs. 1 and 2). Brain magnetic resonance imaging (MRI) showed a massive high signal lesion in his right eye (Fig. 3). The BCVA was 16/200 and IOP was 30 mmHg. Intravenous methylprednisolone, antibiotics, and mannitol were applied with poor response.

The aqueous humor from his right eye was collected. A multiplex PCR/broad-range PCR was applied to screen the pathogen causing the infection.^[4]^ There were no detection of herpes virus species, bacteria, fungi, toxoplasma, toxascaris, or any other pathogens. Next, the chimeric BCR/ABL mRNA and the expression of WT1 mRNA were examined, the details of which are described in the Methods section below.

#### 2.1.1 Detection of major BCR/ABL1 mRNA and WT1 mRNA

The anterior aqueous humor collected from the patient was centrifuged at 2000 × *g* for 5 minutes to harvest cells. RNA was extracted using the RNeasy Micro Kit (Qiagen GmbH, Hilden, Germany). The RNA concentration was measured using a Qubit 3.0 Fluorometer (Thermo Fisher Scientific, Waltham, MA). Complementary DNA was synthesized using the SuperScript IV VILO Master Mix (Thermo Fisher Scientific).^[5]^ Primer sequences for major BCR/ABL1 and WT1 are shown in Table 1. WT1 primers reported by Inoue et al^[3]^ were used. For detection of major BCR/ABL1, the nested polymerase chain reaction (PCR) was performed using AmpliTaq Gold Fast PCR Master Mix (Thermo Fisher Scientific). SimpliAmp Thermal Cycler (Thermo Fisher Scientific) was used for amplification. PCR products were electrophoresed on a 2% agarose gel and analyzed using ChemiDoc XRS+ with Image Lab Software (Bio-Rad, Hercules, CA). For detection of WT1, real-time PCR was performed by LightCycler FastStart DNA Master SYBR Green I (Roche Diagnostics GmbH, Mannheim, Germany). LightCycler 2.0 (Roche Diagnostics GmbH) was used for amplification and analysis.

**Table 1:**
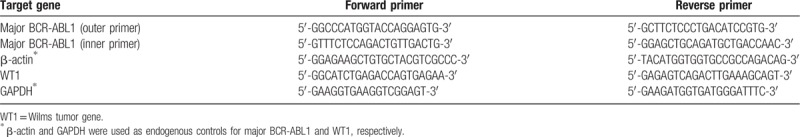
Primer sequences for detecting major BCR-ABL1 and WT1.

**Figure 4:**
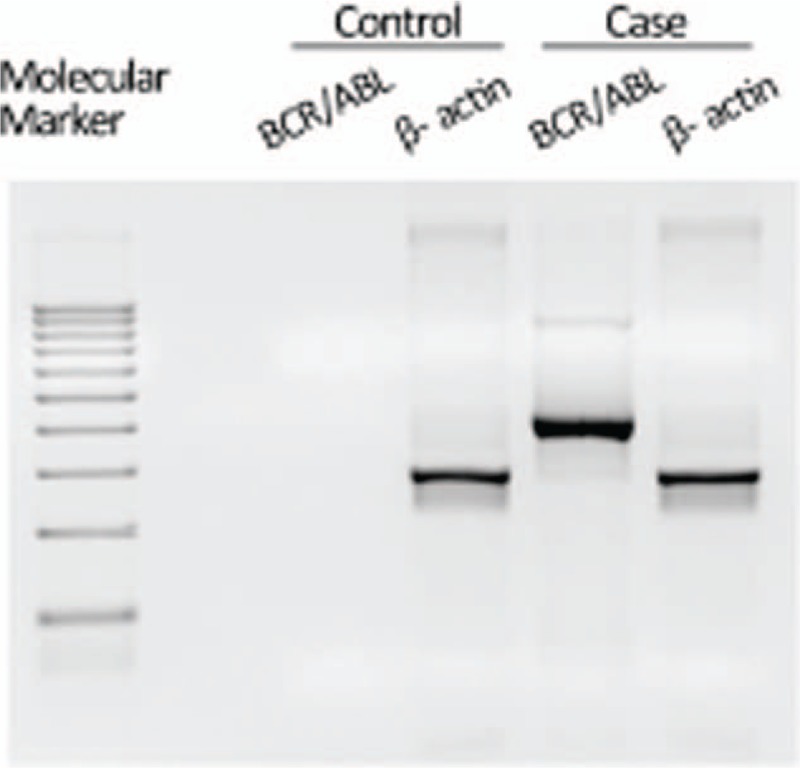
The chimeric BCR/ABL detected in the aqueous humor sample of this case was not found in the normal control peripheral blood. A 100 bp ladder marker as molecular marker was used. The PCR product size of β-actin and chimeric BCR/ABL showed 279 bp and 371 bp, respectively. PCR = polymerase chain reaction.

The chimeric BCR/ABL was detected in the aqueous humor sample of this case (Fig. 4). The aqueous humor from the normal control did not show BCR/ABL chimera by this method (data not shown). The PCR product size in this case with BCR/ABL1 primers was 371 bp. This indicates that the break-points were at the 3′ side of BCR gene exon 13 and 5′ side of ABL gene exon 2, and the translocation pattern was e13a2(b2a2). The same PCR product size had been detected in the patient's bone marrow 15 months earlier (data not shown). The WT1 mRNA expression of the aqueous humor was 2.9 × 10^5^ copies/μg RNA, and that was undetectable in the normal control aqueous humor (data not shown). The bone marrow examination that was performed a week later from aqueous humor paracentesis showed 93,190 copies/μg RNA of BCR/ABL mRNA and 7.1 copies/μg RNA of WT1 mRNA. Neither BCR/ABL nor WT1 mRNA expression was detected in the peripheral blood.

**Figure 5:**
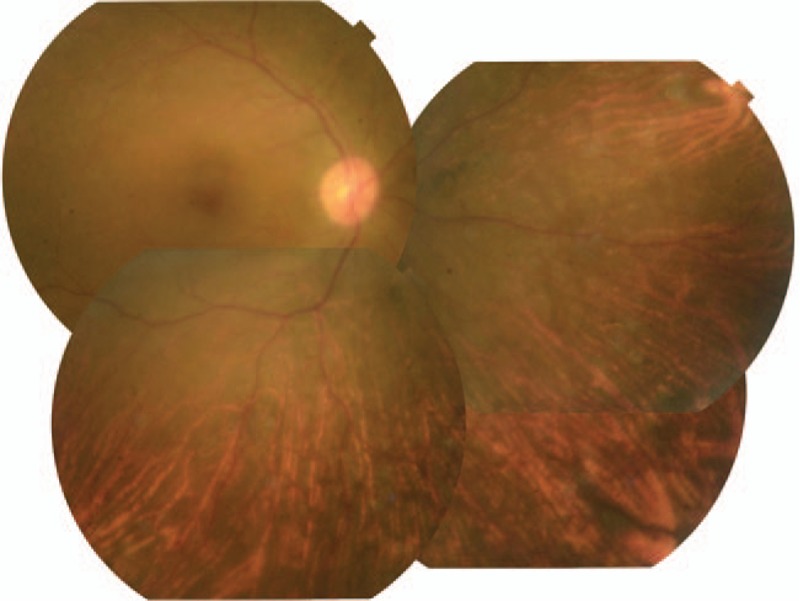
The fundus photographs showed no sign of retinal detachment.

In light of these results, the patient was diagnosed as having a Ph+ALL relapse with intraocular infiltration. Subsequently, dasatinib (100 mg/d) was administered orally, resulting in the hypopyon resolution and IOP normalization (Fig. 5) without any ophthalmological treatment in a week, and retinal detachment was disappeared in 2 months. After 10 months since disanitib administration started, the BCVA of his right eye was 10/200 due to the cataract and retinal degeneration. The patient has continued disanitib therapy for 21 months and showed no recurrance of intraocular infiltration.

## 3 Discussion

In this report, we described a case of leukemic intraocular infiltration developing during the period when the patient was believed to be in remission. The ocular manifestation started as unilateral hypopyon that temporarily responded to topical corticoid application. It then extended to intraocular infiltration with retinal detachment without hematological relapse. The molecular biological examination of the aqueous humor resulted in the diagnosis of Ph+ALL relapse. The ocular manifestation was solely resolved with molecular targeted therapeutics.

The causes of hypopyon vary and include infection, autoimmune diseases, and malignant diseases. The clinical manifestations are similar in resemblance among these causes and it is often difficult to distinguish them solely by ophthalmic examination. The hypopyon is the accumulation of infiltrated inflammatory cells in cases of infection and autoimmune disorders. The intraocular involvement of malignant disease is called masquerade syndrome that resembles uveitis but is resistant to corticosteroid therapy.^[1,6]^ Intraocular infiltration of malignant diseases is thought to occurred through direct infiltration of neoplastic cells, hemorrhage, or by ischemic changes.^[7]^ The phenotype of masqerade syndrome by malignant disease is usually uveitis like but occasionally other phenotypes such as retinal detachment and choroidal thickness are involved.^[1]^ In our case, extensive retinal detachment with high signal lesion in MRI was observed. This suggests the possibility that massive leukemic cells were infiltrated into the subretinal space.

The cause of malignant masquerade syndrome in adults is most often malignant lymphoma and leukemia and, more rarely, metastasis of cancer. It has been reported that 0.2% to 2% of leukemia patients develop leukemic infiltration such as hypopyon in the anterior segment of the eye.^[8–12]^ The etiology of ocular manifestation in leukemia patient is either opportunistic infection following immunosuppressive conditions caused by chemotherapy or leukemic cells infiltration. An accurate diagnosis is essential for precise treatment. In our case, no infectious pathogen was detected by PCR methods. There are several reports indicating intraocular involvement of Ph+ALL.^[13–19]^ Interestingly, in most of these cases, ocular involvement developed during the remission period with normal blood cell counts. Ocular manifestation is commonly diagnosed with blood tests and characteristic ophthalmic findings. However, when the hematological analysis of blood or bone marrow shows less systemic abnormality, intraocular fluid cytology is required. Although topical corticosteroids were not fully effective to hypopyon in our case, there were no other systemic findings. Our oncologist remained uncertain that hypopyon was the sign of relapse until the results of the molecular analysis of the aqueous humor.

There are a few reports in which FISH (fluorescence in situ hybridization) was conducted to detect the BCR/ABL fusion gene in the aqueous humor.^[16–18]^ Although FISH is a popular method to confirm the presence of the chimera gene, it requires a certain amount of cells and false positives can occur through overlap. Therefore, we decided to perform PCR, and were able to determine the BCR/ABL fusion gene consistent with the diagnosis of Ph+ALL. The eye is a small organ, and there are few tissues and samples, including intraocular fluid, that are safe to procure for examination. The PCR method is beneficial for small sample volumes and rapidity in noting results. Similar to our case, when there is no evidence of leukemia in the blood, molecular biological examination of intraocular fluid is favorable. These results led us to perform a bone marrow examination and ultimately, we were able to confirm relapse. There are several additional reports in which ocular involvement was the single sign of leukemic relapse, similar to our case.^[10,15,16,18–21]^

The WT1 gene was initially identified as a tumor suppressor gene, and the mutation of the gene led to the development of pediatric renal tumors. It was then determined that WT1 was overexpressed in the majority of acute meloid leukemia (AML) and ALL cases.^[22]^ Additionally, the increase of WT1 expression was concomitant to the progression of chronic myeloid leukemia (CML) and myelodysplastic syndromes (MDS).^[3,23,24]^ The expression level reflects the activity of leukemia that is independent from chimiric DNA. WT1 is thought to be one of the markers for leukemia, and that can be used for predicting prognosis and monitoring relapse. Recently, WT1 peptide vaccination have demonstrated positive survival efficacy on leukemia patients.^[25–27]^ We have examined WT1 mRNA expression of the aqueous humor. Surprisingly, the expression was much higher than that of bone marrow during the same period, 2.9 × 10^5^ copies/μg and 7.1 copies/μg of RNA respectively. It was also undetactable in the control aqueous humor. These results suggest that monitoring WT1 expression in intraocular fluid can be a good candidate marker to screen leukemic infiltration in the eye regardless of leukemia subtype. In addition, it appears that the extent of WT1 expression in the eye reflects the leukemic infiltration severity even under silent phenotypic general conditions.

The present findings suggest the importance of prompt diagnosis of leukemic infiltration in the eye using molecular biological techniques. Furthermore, it raises the possibility of WT1 expression as the marker for topical leukemia relapse.

## Author contributions

**Conceptualization:** Miki Hiraoka.

**Data curation:** Miki Hiraoka, Hiroshi Ikeda, Daisuke Furuya.

**Formal analysis:** Daisuke Furuya.

**Investigation:** Miki Hiraoka, Hiroshi Ikeda.

**Methodology:** Daisuke Furuya.

**Project administration:** Satoshi Takahashi.

**Resources:** Miki Hiraoka.

**Supervision:** Hiroshi Ohguro.

**Writing – original draft:** Miki Hiraoka.

**Writing – review & editing:** Hiroshi Ikeda, Daisuke Furuya, Satoshi Takahashi.

Miki Hiraoka orcid: 0000-0002-6411-2269.

Hiroshi Ohguro orcid: 0000-0003-1052-0280.
